# The Concept about the Regeneration of Spent Borohydrides and Used Catalysts from Green Electricity

**DOI:** 10.3390/ma8063456

**Published:** 2015-06-10

**Authors:** Cheng-Hong Liu, Bing-Hung Chen

**Affiliations:** 1 Division of New Energy System, Green Energy and Eco-technology System Center, Industrial Technology Research Institute (ITRI)-South Campus, Tainan 73445, Taiwan; E-Mail: ch_liu@itri.org.tw; 2 Department of Chemical Engineering, National Cheng Kung University, Tainan 70101, Taiwan

**Keywords:** Brown-Schlesinger process, sodium borohydride (NaBH_4_), ammonia borane (NH_3_BH_3_), hydrogen storage, sodium hydride (NaH), used catalysts, green electricity

## Abstract

Currently, the Brown-Schlesinger process is still regarded as the most common and mature method for the commercial production of sodium borohydride (NaBH_4_). However, the metallic sodium, currently produced from the electrolysis of molten NaCl that is mass-produced by evaporation of seawater or brine, is probably the most costly raw material. Recently, several reports have demonstrated the feasibility of utilizing green electricity such as offshore wind power to produce metallic sodium through electrolysis of seawater. Based on this concept, we have made improvements and modified our previously proposed life cycle of sodium borohydride (NaBH_4_) and ammonia borane (NH_3_BH_3_), in order to further reduce costs in the conventional Brown-Schlesinger process. In summary, the revision in the concept combining the regeneration of the spent borohydrides and the used catalysts with the green electricity is reflected in (1) that metallic sodium could be produced from NaCl of high purity obtained from the conversion of the byproduct in the synthesis of NH_3_BH_3_ to devoid the complicated purification procedures if produced from seawater; and (2) that the recycling and the regeneration processes of the spent NaBH_4_ and NH_3_BH_3_ as well as the used catalysts could be simultaneously carried out and combined with the proposed life cycle of borohydrides.

## 1. Introduction

With a rapid increase in population worldwide, demand for energy, mostly from combustion of fossil fuels, is raised dramatically. Consequently, the atmospheric concentrations of several greenhouse gases such as carbon dioxide (CO_2_), nitrous oxide (N_2_O), and methane (CH_4_) have increased by around 40% since the large-scale industrialization began in the early 19th century, leading to global warming and severe climate change. In addition, soot and fine particulate matters associated with the combustion of fossil fuels have been released into the atmosphere every year and have thus threatened the lives of many humans. Furthermore, the ecological balance on earth will also be destroyed if such problems are not resolved as early as possible. Therefore, the research and development of sustainable energy have been extensively carried out worldwide.

Hydrogen energy plays quite an important role in terms of sustainable energy. For example, the hydrogen gases can react with oxygen to generate electric power in fuel cells. Alternatively, the fuel cell can directly convert chemical energy into electricity, accompanied with the generation of water and heat. Among various types of fuel cells, the proton-exchange-membrane fuel cell (PEMFC) possesses some attractive advantages, for instance, lower operating temperature, more compact, lower weight, sustained operation at a higher current density, longer stack life, quicker start-up, suitability to discontinuous operation, and potential for a lower operation cost and volume [1]. However, for the longer operation duration of PEMFC, a stable supply of ultrapure hydrogen is necessary. Therefore, the development of an applicable hydrogen storage and supply technology attracts enormous attention.

No viable hydrogen storage method has been commercialized so far owing to many limitations such as the size, weight and cost. To overcome the barriers mentioned above, the hydrogen supplied to the PEMFC would be preferentially produced on site and on demand. In addition to the hydrogen production techniques, other topics such as an efficient distribution and storage of the hydrogen should be taken into account as well in order to realize the hydrogen economy. For example, the hydrogen storage and supply is a critical issue in the automobile on-board system, on which a higher volumetric and gravimetric energy density but the smaller size and the less weight of the container for the hydrogen storage are desired.

Among various H_2_ storage methods, chemical hydride fuel systems, composed of lighter elements than metal hydrides, exhibit the higher gravimetric hydrogen density. Release of hydrogen from chemical hydrides can be mostly divided into two ways, inclusive of pyrolysis reaction and hydrolysis method. The representative reactions of the hydrolysis reaction are shown in the Equations (1) and (2).

*MH_y_ + y H_2_O →M(OH)_y_ + y H_2_* (1)

where *M* is a metal and *y* is the valence of metal [2].

*MXH_4_ + 4 H_2_O → 4 H_2_ + MOH +H_3_XO_3_* (2)

where *M* is an alkali metal (Group IA), and *X* for a trivalent element from Group IIIA [2].

Chemical hydrides such as LiBH_4_, NaBH_4_, KBH_4_, LiH, NaH, and MgH_2_ can react with water at an ambient condition with/without the presence of suitable catalysts, leading to the generation of hydrogen of high purity with no CO evolved, which can be directly fed into the fuel cell system without any prior purification required. In contrast, the hydrogen gas produced from the steam reforming methods contains inevitably a significant level of CO and has to undergo the purification measure before being consumed in the PEMFCs. Notably, the metal borohydrides are considered the better candidates for the hydrogen storage system because of their high storage capacity [3]. Moreover, among different metal borohydrides, NaBH_4_ is regarded as one having a higher potential to meet the goals declared by the US DOE because of its higher hydrogen content (*i.e*., 10.8 wt%) through the hydrolysis reaction as follows [4]:

(3) $$NaBH_{4}+2H_{2}O\overset{\;catalyst\;}{\to }NaBO_{2}+4H_{2}+217kJ$$

Nevertheless, with relentless effort numerous works have been conducted in developing suitable catalysts to promote the H_2_ generation from aqueous NaBH_4_ systems. Still, existence of the intrinsic defects in the aqueous NaBH_4_ systems, such as poor aqueous solubility of spent borohydride—metaborate—restricts their progress in practical applications. For example, instead of the ideal condition presented in Equation (3), the poorer aqueous solubility of the sodium metaborate, produced along with the evolution of the hydrogen gas from the hydrolysis of the NaBH_4_ shown in Equation (3), often results in solid precipitates on catalysts, blocking the active catalytic sites for the subsequent hydrolysis reaction of NaBH_4_ to produce hydrogen. Furthermore, not all hydrogen atoms in water molecules involved in the hydrolysis of NaBH_4_ are transformed into the produced hydrogen gas. Instead, many of them are rather taken up by the metaborate molecules to form various types of hydrates, as shown in Equation (4) [5], and, hence, the gravimetric storage density of the hydrogen in such a chemical hydride fuel system is decreased.

(4) $$NaBH_{4}+(2+x)H_{2}O\overset{\;catalyst\;}{\to }NaBO_{2}\cdot xH_{2}O+4H_{2}$$

As a result, the hydrogen storage capacity in an aqueous NaBH_4_ system would generally decrease from theoretically predicted 10.8 wt% to 7.5 wt%, when the aqueous solubility of NaBH_4_, *i.e*., 55 g NaBH_4_ per 100 g H_2_O or equivalently 35.48 wt% at 25 °C, is considered. Furthermore, if taking the aqueous solubility of NaBO_2_, *i.e*., 28 g NaBO_2_ per 100 g H_2_O at 25 °C, into account, NaBO_2_ becomes a limiting reagent in the hydrolysis reaction of NaBH_4_ for hydrogen generation. Consequently, the initial NaBH_4_ concentration in the hydrogen generation system should not exceed 16 g NaBH_4_ per 100 g H_2_O at 25 °C in order to maintain a liquid state during the whole course of hydrogen production. Otherwise, the excess NaBO_2_ produced would precipitate out on the catalyst surface and, hence, could seriously reduce the performance of hydrogen generation by deactivating the active sites of catalysts [5]. Accordingly, the hydrogen storage capacity in an aqueous NaBH_4_ system would further be decreased down to 2.9 wt% or even lower if any hydrated NaBO_2_ is present. This is also one of the major reasons that the no-go decision of using NaBH_4_ aqueous system for the onboard hydrogen storage was made by the US DOE in 2007, according to the test reports from Millennium Cell, ANL and TIAX laboratory [6]. The other reason attributable to the No-Go decision is the high-energy penalty and cost of regenerating sodium metaborate (NaBO_2_) back to NaBH_4_ fuel [6]. That is, the hydrogen cost and energy efficiency are of significant concern and need to be overcome to witness the realization of the chemical hydride fuel system.

In order to reduce the cost of the hydrogen evolved from the NaBH_4_ fuel systems, a concept of utilizing water, instead of borohydrides, as a limiting agent in hydrogen production was recently proposed [7,8]. In brief, the presence of excess water has increased the total mass of the system and, thus, has reduced the total gravimetric and volumetric capacities of the hydrogen stored in such NaBH_4_ fuel systems. That is, the hydrogen production from such a NaBH_4_ hydrogen storage system mainly takes place in the solid phase or in the liquid phase in close proximity to the solid-liquid boundary. Therefore, the gravimetric storage capacity of hydrogen in the NaBH_4_/H_2_O system could be effectively enhanced. Liu *et al*. [7] and Gislon *et al*. [8] both pointed out that the effective H_2_ storage capacities as high as 6.7 wt% and 6.5 wt%, respectively, could be achieved in such a solid NaBH_4_ hydrogen storage system. Moreover, the gravimetric hydrogen storage capacity was further improved to *ca*. 7.3 wt% from the solid-state NaBH_4_/Ru-based catalyst composites prepared from a high-energy ball-milling process [9]. This also implies that a superior H_2_ storage capacity from the solid NaBH_4_ hydrogen storage systems could possibly reach the set 2010 target of US DOE at 6 wt% for on-board system.

In addition to the on-board applications, NaBH_4_ has been attempted to be applied to portable devices or the maintenance-free stationary systems, for instance, to be used in a cell phone charger, and to feed the fuel cells in the off-grid remote warning system because of its long shelf life. As aforementioned, besides the issue of the hydrogen storage capacity, the cost of NaBH_4_ and the difficulty in recycling the spent-NaBH_4_, *i.e*., NaBO_2_, back to the borohydride fuel are the main causes leading to the No-Go recommendation to the NaBH_4_-based hydrogen storage system for the vehicle on-board applications [6]. That is, the production cost of NaBH_4_ is still posting a hurdle to its practical applications. In the subsequent sections of this report, the life cycle of the NaBH_4_ is discussed in order to shed light on the strategy in alleviation of the production cost and recycling of the NaBH_4_.

In order to increase the hydrogen storage capacity, NH_3_BH_3_ (commonly denoted as AB) has become the center of recent relevant studies [10,11,12,13], especially after the US DOE’s No-Go recommendation to NaBH_4_ for on-board automotive hydrogen storage [5,6]. NH_3_BH_3_ (borazane) contains intrinsically 19.6 wt% hydrogen. Notably, the hydrogen storage capacity of NH_3_BH_3_ is generally accepted to possibly reach more than 9 wt%. More importantly, NH_3_BH_3_ and its spent products after hydrogen released via the hydrolysis reaction is rarely toxic, stable and easily handled at ambient conditions [10,11,12,13].

NH_3_BH_3_ is generally known to possess many advantages: (1) its long-term storage stability, for example, more than 80 days stable in aqueous solution under an argon atmosphere [14], and (2) the smallest volume occupied for hydrogen supply, e.g., 13.32 mL for NH_3_BH_3_ in relative to 17.38 mL for NaBH_4_ and 51.11 mL for compressed hydrogen at 70 MPa and 288 K to supply one mole of hydrogen. Explicitly, this makes NH_3_BH_3_ suitable as a hydrogen storage medium for on-board vehicle applications. In addition, hydrogen in great purity can be obtained from the hydrolysis reaction of NH_3_BH_3_ in presence of particular catalysts, including noble metals [15,16], transition metals [14,17], even acids and carbon dioxide [10]. Alternatively, hydrogen can be liberated through the pyrolysis of NH_3_BH_3_ between 137 and 400 °C as well [14,18].

## 2. Life Cycle of Sodium Borohydride (NaBH_4_) and Ammonia Borane (NH_3_BH_3_)

**Figure 1:**
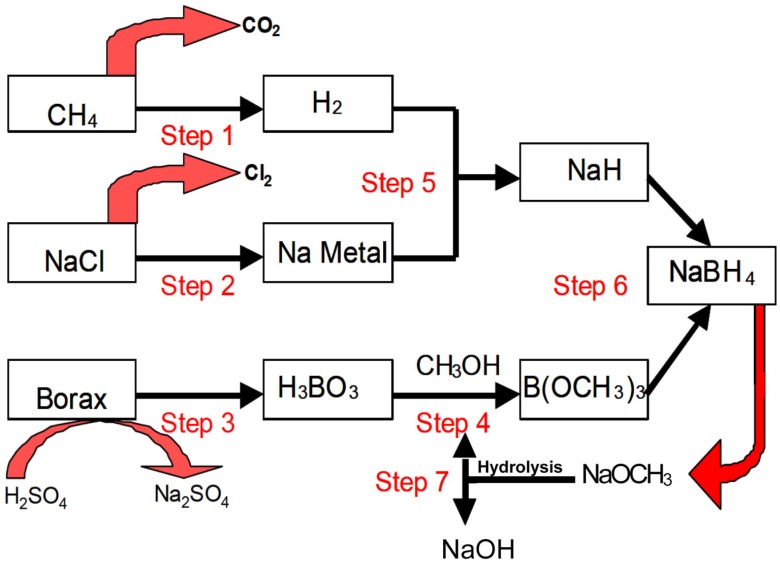
The Brown-Schlesinger process for the synthesis of NaBH_4_ [19].

The prevailing production method of NaBH_4_ in industrial scale is the Brown-Schlesinger process [4], which generally consists of seven steps [19] that are schematically shown in Figure 1 and described briefly as follows:

Step 1. Hydrogen produced from steam reforming of methane.
Step 2. Metallic sodium obtained through the electrolysis of sodium chloride.
Step 3. Boric acid converted from borax.
Step 4. Trimethyl borate synthesized from esterification of boric acid in methanol.
Step 5. Sodium hydride produced from metallic sodium reacting with hydrogen.
Step 6. Synthesis of NaBH_4_ via the reaction of trimethyl borate with sodium hydride.
Step 7. Methanol recycled from the hydrolysis of sodium methoxide.

The large-scale synthesis of NH_3_BH_3_ was first reported by Shore and Böddeker in 1964 [20]. In brief, NH_3_BH_3_ was obtained by extraction with ether from the solid mixture resulted from diborane dispersed in tetrahydrofuran (THF) passed with ammonia at −78 °C, close to the melting point of ammonia at −77.73 °C [20].

(5) $$THF\cdot BH_{3}+NH_{3}\overset{THF}{\underset{\;-78{}^{\circ}C\;}{\to }}NH_{3}BH_{3}+THF$$

Recently, a simplified process close to the ambient condition is devised to synthesize NH_3_BH_3_ from NaBH_4_ and ammonia sulfate in tetrahydrofuran at 40 °C shown as Equation (6), in contrast to −78 °C for the Shore-Böddeker process [20].

(6) $$2NaBH_{4}+{\left(NH_{4}\right)}_{2}SO_{4}\overset{THF}{\underset{\;40{}^{\circ}C\;}{\to }}2NH_{3}BH_{3}+Na_{2}SO_{4}+2H_{2}$$

Nonetheless, the NH_3_BH_3_ is still too expensive for practical applications. For example, according to the information supplied from Sigma-Aldrich, the current price tag for the NH_3_BH_3_ in technical grade with a purity of 90% is US$ 158.50 per 10 g, in contrast to US$ 201 per 500 g for NaBH_4_ with a purity >96%. Moreover, in view of NH_3_BH_3_ as a sustainable supply of hydrogen, it is desirable to regenerate the spent product of NH_3_BH_3_ after hydrogen production.

**Figure 2:**
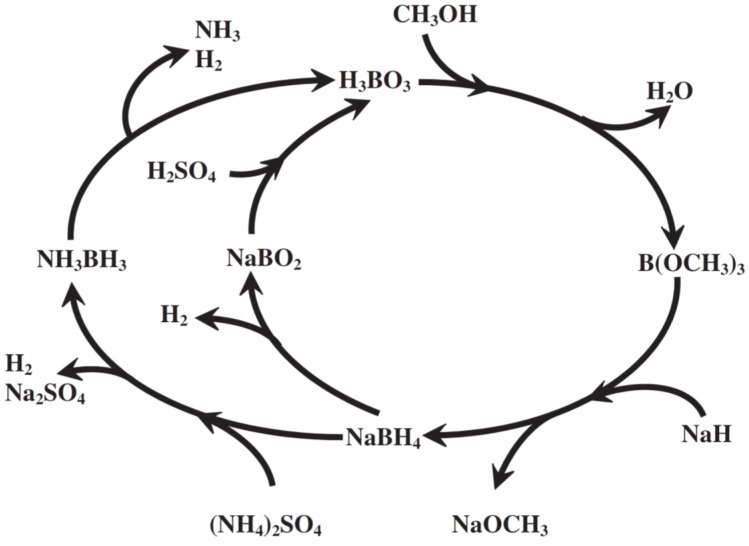
The proposed total life cycle of borohydrides for hydrogen generation [22].

Previously, boric acid (H_3_BO_3_) was found to be one major product in the hydrolysate of NH_3_BH_3_ [21]. Boric acid is likely resulted from the quick acidification of sodium metaborate (NaBO_2_) in the presence of H_2_SO_4_ or in acidic condition [21]. Notably, boric acid is one of the reactants used in the production of trimethyl borate (B(OCH_3_)_3_), a precursor of the NaBH_4_ through the Brown-Schlesinger process [22]. Subsequently, NaBH_4_ could be fabricated by reacting trimethyl borate with sodium hydride (NaH) at 220–250 °C in oil bath with the Brown-Schlesinger process [4]. Thus, the total life cycle between NH_3_BH_3_ (NH_3_BH_3_) and NaBH_4_ for hydrogen generation could be sketched (Figure 2), which illustrates the possible pathways in hydrogen production from these chemical hydrides. Furthermore, with the information shown in Figure 2, the possible regeneration scheme of these spent chemical hydrides harvested from those after hydrogen evolution can be explored and investigated to shed a light to the realization of hydrogen economy by reducing the production cost of the NH_3_BH_3_. That is to say, once the cost of NaBH_4_ could be reduced, less expensive NH_3_BH_3_ could be manufactured.

Among various production steps in the Brown-Schlesinger process, the one consuming the most energy is undoubtedly the electrolysis of NaCl to produce metallic sodium. The NaCl come from evaporation of seawater in the saltern, where the offshore wind farm is closely located. Moreover, metallic sodium is so active that occupies high cost during transportation with great care. Therefore, the *in-house* production of metallic sodium and sodium hydride will benefit the reduction in the production cost of NaBH_4_ as well as NH_3_BH_3_.

**Figure 3:**
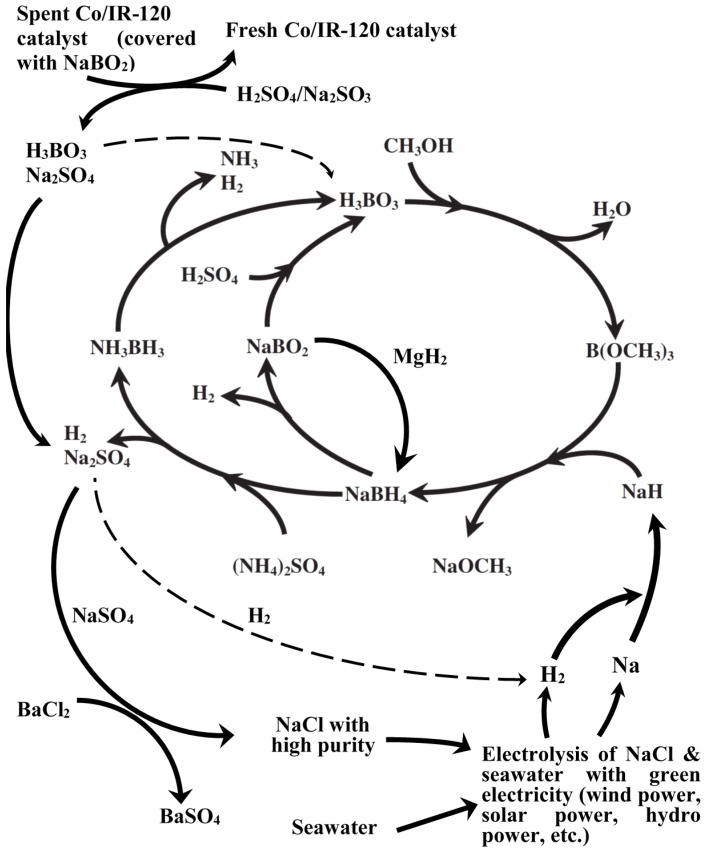
The modified total life cycle of borohydrides for hydrogen generation.

In this report, we will provide several possible solutions for the reduction in the production cost and the hazard and risk in the Brown-Schlesinger process either by the combination of green energy (*i.e*., offshore wind power) and the electrolysis from seawater for the localized production of H_2_ and NaH. The revised life cycle of NaBH_4_-NH_3_BH_3_ will be presented accordingly in Figure 3 and discussed in the following section.

## 3. Modified Life Cycle of Sodium Borohydrides (NaBH_4_) and Ammonia Borane (NH_3_BH_3_)

As mentioned above, the practice to fabricate metallic sodium from the electrolysis reaction of NaCl takes up the highest cost in the commercial Brown-Schlesinger process. Within this step, the use of the extensive amount of electricity in the electrolysis is the main factor attributable to the large production cost. Hence, it is possible to reduce such a production cost with green electricity generated from an offshore wind farm [23]. Briefly, 1.8 GWh is in need when performing the electrolysis of the seawater of 100,000 ton/day (assuming the electrolysis efficiency near 42%). Taking the average resale price of the electricity around US$0.12/kWh into account, US$2.16 million could be saved when processing the seawater of 100,000 tons to produce 1000 tons of sodium metal everyday by using the offshore wind power electricity. In addition, the hydrogen generated during the electrolysis of the NaCl solution could be further conducted to react with the obtained metallic sodium metal to produce NaH on-site. Sodium hydride is regarded as a more stable product than the metallic sodium and is more advantageous for a safer and less costly transportation.

Based on the idea using the green electricity generated from the offshore wind power as a feasibly economic measure to reduce the production cost of sodium metal and sodium hydride, we have modified the total life cycle of NH_3_BH_3_ and NaBH_4_ previously published in the open literature [21] (Figure 3). Also, the strategy for the regeneration of the used Co catalyst is duly proposed and integrated into the revised life cycle (Figure 3).

In the synthesis of NH_3_BH_3_, NaBH_4_ first reacts with (NH_4_)_2_SO_4_ in THF at 40 °C to give NH_3_BH_3_ with hydrogen gas and Na_2_SO_4_ as the byproducts, as depicted in Equation (6). The produced hydrogen gas has to be collected and can be applied to the production process of sodium hydride (Na+½H_2_→NaH). On the other hand, Na_2_SO_4_ solution could contact with BaCl_2_ to have BaSO_4_ precipitate out of the aqueous reagent system, which is presumably dominated by NaCl. The NaCl solution with a high purity can be directly electrolyzed with the green electricity generated from the offshore wind power electricity. The other byproduct, BaSO_4_, is known to possess a high commercial value and can serve as an oil-well drilling fluid, a white pigment, a paper brightener, a plastics filler, a contrast agent, *etc.* These valuable byproducts shall be able to offset the high production cost of NaBH_4_ and a NH_3_BH_3_.

## 4. Recycle and Regeneration of Used Cobalt Catalysts

**Figure 4:**
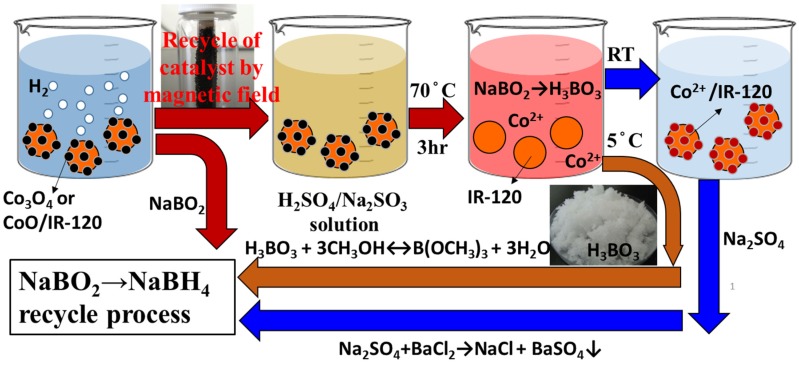
The combined concept of the regeneration processes of the spent-NaBH_4_ and the spent catalyst.

The used catalysts are recycled and regenerated not only for the sake of the economics consideration but also the sustainability. Previously, the magnetic cobalt catalysts loaded on the resin beads (Co/IR-120) have been successfully synthesized and applied to catalyze the hydrolysis of NaBH_4_ in alkaline solution for hydrogen evolution [24]. Not only a high production rate of hydrogen, but also the easiness in the recovery of the used Co/IR-120 from reacting system with permanent magnet was achieved [24]. The surface chemistry of the prepared Co/IR-120 catalysts, made from the reduction of chelated cobalt ions on polymer resin by NaBH_4_, was found mainly as cobalt oxides (Co_3_O_4_ and CoO), but not cobalt borides (Co_2_B), according to the XPS analyses [24]. With this information, a recycling process of the spent Co/IR-120 catalysts was devised for the simultaneous recycling and regeneration process to the spent-NaBH_4_ and the used catalysts (Figure 4).

In general, NaBO_2_ is one major component of the spent-NaBH_4_ and has a relatively lower solubility, *i.e*., 28 g in 100 mL H_2_O at 25 °C, than NaBH_4_. Thus, NaBO_2_ will precipitate on the surface of catalysts after the long-term hydrolysis reaction of NaBH_4_ in concentrated solutions, leading to the deactivation of the catalyst. Notably, CoO could dissolve easily in H_2_SO_4_ solution (Equation (7)). However, the solubility of Co_3_O_4_ was much lower than that of CoO because Co_3_O_4_ is more stable than CoO [25]. Fortunately, this problem can be resolved with the assistance of sulfite (SO_3_^2−^) that will facilitate the conversion of Co^3+^ to Co^2+^. The reduction-dissolution of Co_3_O_4_ can be presented as Equation (8), accordingly.

(7) $$\text{CoO}+{\text{H}}_{2}{\text{SO}}_{4}\to \text{CoS}{\text{O}}_{4}+{\text{H}}_{2}\text{O}$$

(8) $$\text{C}{\text{o}}_{3}{\text{O}}_{4}+3{\text{H}}_{2}\text{S}{\text{O}}_{4}+\text{N}{\text{a}}_{2}\text{S}{\text{O}}_{3}\to 3\text{CoS}{\text{O}}_{4}+\text{N}{\text{a}}_{2}\text{S}{\text{O}}_{4}+3{\text{H}}_{2}\text{O}$$

Consequently, the used Co/IR-120 catalyst after the hydrolysis reaction of NaBH_4_ could be recycled conveniently with magnets and, then, immersed in the H_2_SO_4_/Na_2_SO_3_ solution at an appropriate concentration and temperature (*i.e*., 70 °C for 3 h), shown in Figure 4. During the immersion procedure, Co^2+^ ion will be leached out from the surface of Co/IR-120 catalyst, while H_3_BO_3_ can be simultaneously obtained from NaBO_2_ precipitated on catalyst with acids (Equation (9)).

(9) $$2NaBO_{2}\cdot 4H_{2}O+H_{2}SO_{4}\overset{\;70{}^{\circ}C\;}{\underset{\;3h\;}{\to }}2H_{3}BO_{3}+Na_{2}SO_{4}+6H_{2}O$$

The boric acid could be easily separated from the reacting mixture by crystallization at a lower temperature. For example, being cooled to 5 °C, needle-like H_3_BO_3_ crystal appears and precipitates out from the coexisting Na_2_SO_4(aq)_ [22]. Thus, boric acid could be conveniently collected through filtration. The boric acid will be, subsequently, esterified with excess methanol to yield trimethyl borate [22], which can be further converted to NaBH_4_ with NaH, according to the commercial Brown-Schlesinger process. Besides, the fresh Co^2+^ ions can be chelated with the benzenesulfonyl groups on the IR-120 ion exchange resin beads again to generate the fresh Co/IR-120 catalysts to complete the regeneration process of the used catalyst.

The rest left in the solution after filtering-out H_3_BO_3_ crystals is dominantly the Na_2_SO_4_. BaCl_2_ can be introduced to precipitate out BaSO_4_ and to leave NaCl in the solution phase (Figure 3). Finally, Na metal and hydrogen gas can be obtained from the electrolysis of the NaCl solution with the green electricity, Namely, the introduction of the green electricity to the recycling and regeneration processes of the spent borohydrides and NH_3_BH_3_ as well as the used catalysts could increase the sustainability of the chemical hydride H_2_-fuel system and, therefore, realize the hydrogen economy.

## 5. Conclusions

In this report, we have proposed our modified life cycle of NaBH_4_ and NH_3_BH_3_, based on the concept of combining the use of green energy, such as green electricity generated from an offshore wind farm, instead of the electricity currently and majorly generated from the fossil fuels, to produce sodium hydride and, subsequently, to manufacture NaBH_4_ and NH_3_BH_3_. Consequently, the production costs of metallic sodium and sodium hydride are always the most expensive in the commercial Brown-Schlesinger process for the production of NaBH_4_, and could be saved with green electricity generated *in situ*. The NH_3_BH_3_ can be further synthesized from NaBH_4_ recycled from the spent chemical hydrides. The spent Co catalysts could be recycled and regenerated with the use of sulfuric acid and sodium sulfite. That is, the recycling and the regeneration processes of the spent NaBH_4_ and NH_3_BH_3_ as well as the used catalysts could be simultaneously carried out and combined with the proposed life cycle of borohydrides. Thus, the total efficiency, both in energy and materials, of the used catalysts and the spent chemical hydrides could be greatly improved. Notably and alternatively, the main differences and advances of the life cycle proposed in this study from that published in the literature is on the use of green energy and electricity, instead of that supplied from a fossil-fuel power station, and that the regeneration of the spent chemical hydrides and the used catalysts could be performed in one cycle with a possibly higher efficiency.
